# Huntingtin is required for ER-to-Golgi transport and for secretory vesicle fusion at the plasma membrane

**DOI:** 10.1242/dmm.017368

**Published:** 2014-10-31

**Authors:** Hemma Brandstaetter, Antonina J. Kruppa, Folma Buss

**Affiliations:** 1 Cambridge Institute for Medical Research, University of Cambridge, Cambridge, CB2 0XY, UK.

**Keywords:** Exocytosis, Huntingtin, ER, Golgi, Vesicle fusion

## Abstract

Huntingtin is a large membrane-associated scaffolding protein that associates with endocytic and exocytic vesicles and modulates their trafficking along cytoskeletal tracks. Although the progression of Huntington’s disease is linked to toxic accumulation of mutant huntingtin protein, loss of wild-type huntingtin function might also contribute to neuronal cell death, but its precise function is not well understood. Therefore, we investigated the molecular role of huntingtin in exocytosis and observed that huntingtin knockdown in HeLa cells causes a delay in endoplasmic reticulum (ER)-to-Golgi transport and a reduction in the number of cargo vesicles leaving the trans-Golgi network. In addition, we found that huntingtin is required for secretory vesicle fusion at the plasma membrane. Similar defects in the early exocytic pathway were observed in primary fibroblasts from homozygous *Htt^140Q/140Q^* knock-in mice, which have the expansion inserted into the mouse huntingtin gene so lack wild-type huntingtin expression. Interestingly, heterozygous fibroblasts from a Huntington’s disease patient with a 180Q expansion displayed no obvious defects in the early secretory pathway. Thus, our results highlight the requirement for wild-type huntingtin at distinct steps along the secretory pathway.

## INTRODUCTION

Huntington’s disease (HD) is an autosomal dominant neurodegenerative disorder that is caused by a polyglutamine (polyQ) repeat expansion at the N-terminus of the protein huntingtin (HTT) (DiFiglia et al., 1997; Imarisio et al., 2008). The age of disease onset and the rate of aggregation depend on the length of the polyQ expansion; for example, 40–50 repeats cause adult-onset HD, whereas a large number of repeats (e.g. 180) leads to juvenile forms of the disease (Squitieri et al., 2002; Snell et al., 1993). HD progression is complex and clearly involves a gain of toxic functions of the mutant protein leading to the formation of protein aggregates, the accumulation of damaged mitochondria, proteasome and autophagy dysfunction, and finally neuronal cell death (Borrell-Pagès et al., 2006). Hence, a detailed analysis of the diverse cellular functions of wild-type huntingtin is required to understand the exact contribution(s) that loss of wild-type huntingtin plays in disease progression (Schulte and Littleton, 2011; Caviston and Holzbaur, 2009).

In mammals, loss of wild-type huntingtin is embryonic lethal, highlighting the essential cellular role of this protein (Nasir et al., 1995; Duyao et al., 1995; Zeitlin et al., 1995). Huntingtin is a large scaffold protein that associates with a variety of cellular binding partners (Shirasaki et al., 2012), which mediate huntingtin’s multiple roles in intracellular transport and membrane trafficking (Caviston and Holzbaur, 2009). For example, huntingtin-associated protein 1 (HAP1) links huntingtin to both plus-end-directed kinesin and minus-end-directed dynein motor proteins, emphasising the importance of huntingtin for anterograde and retrograde axonal transport along microtubule tracks (Li et al., 1995; Li and Li, 2005; Engelender et al., 1997). Furthermore, huntingtin can also associate through optineurin with the actin-based motor myosin VI, and thus huntingtin is in the unique position to coordinate transport along the microtubule and actin cytoskeleton (Hattula and Peränen, 2000; Sahlender et al., 2005).

Transport defects become noticeable during HD progression when reduced levels of brain-derived neurotrophic factor (BDNF) are transported and secreted by cortical neurons and astrocytes. Consequently, less BDNF is taken up by striatal neurons, and this might play an important role in the selective neuronal vulnerability and degeneration associated with this disease (Wang et al., 2012). Thus, in this study, we have used a novel fluorescent reporter assay to investigate the molecular role of huntingtin in protein secretion using HeLa cells expressing *HTT* siRNA and homozygous primary embryonic fibroblasts isolated from the *Htt^140Q/140Q^* knock-in (KI) mouse as well as primary fibroblasts from an individual with HD who is heterozygous for 180Q at the *HTT* locus (*HTT^+/180Q^*). Our results demonstrate that wild-type huntingtin is required in the secretory pathway in HeLa cells for vesicle transport from the endoplasmic reticulum (ER) to the Golgi complex, for the formation and delivery of vesicles from the Golgi to the plasma membrane, and for vesicle fusion with the plasma membrane. Comparing protein secretion in *HTT*-siRNA-treated cells, homozygous *Htt^140Q/140Q^* mouse embryonic fibroblasts (MEFs) and heterozygous *HTT^+/180Q^* patient fibroblasts suggests that one copy of wild-type huntingtin is sufficient to maintain delivery of secretory cargo between the ER and the Golgi complex in fibroblasts. Interestingly, secretion is not affected by the toxic gain of function of mutant huntingtin protein in heterozygous patient fibroblasts.

## RESULTS

### Huntingtin and dynein function is required in the early secretory pathway

To probe the role(s) of wild-type huntingtin in the secretory pathway, we used a HeLa cell line (termed HeLa C1) stably expressing a constitutively secreted GFP-tagged variant of the human growth hormone (hGH). This reporter construct (GFP-hGH) is attached to multiple aggregation domains, which cause the newly synthesised GFP-hGH protein to aggregate and be retained in the ER. Treating the cells with a small ligand (AP21998) reverses the aggregation, resulting in a synchronous release of GFP-hGH from the ER to the Golgi complex and then on to the plasma membrane for exocytosis (Bond et al., 2011; Gordon et al., 2010). This tightly regulated secretion assay provides an elegant method for visualising these main stages of constitutive secretion by live-cell time-lapse fluorescence microscopy.

**TRANSLATIONAL IMPACT**

**Clinical issue**

Huntington’s disease (HD) is an incurable, autosomal dominant neurodegenerative condition characterised by late-onset motor, cognitive and psychiatric symptoms. HD is linked to mutations in the *HTT* gene that lead to an extended polyglutamine repeat at the N-terminus of the huntingtin protein. This mutant protein is prone to aggregation and is thereby cytotoxic for neurons, leading to neuronal loss in the striatum and cortex. The huntingtin protein is ubiquitously expressed in the brain and has multiple roles in cellular membrane trafficking, including anterograde axonal transport of neurotrophic factors [such as brain-derived neurotrophic factor (BDNF)] for release from cortical neurons. During HD progression, transport defects become noticeable, with decreased secretion of BDNF by cortical neurons and astrocytes, and consequently reduced uptake of BDNF by striatal neurons; this might play an important role in the selective neuronal vulnerability and degeneration associated with this disease.

**Results**

To investigate the role of wild-type huntingtin in protein secretion, the authors use a combination of tissue culture cell lines, including primary fibroblasts isolated from homozygous *Htt^140Q/140Q^* knock-in mice, *HTT*-knockdown human HeLa cells and primary fibroblasts obtained from a heterozygous individual with HD (*HTT^+/180Q^*). They use a unique secretory assay that allows dissection of the exact step(s) that require the huntingtin protein during protein secretion. The authors demonstrate that wild-type huntingtin is required for vesicle transport from the endoplasmic reticulum (ER) to the Golgi complex, for the formation and delivery of vesicles from the Golgi to the plasma membrane, and for vesicle fusion with the plasma membrane. The complete loss of wild-type huntingtin in homozygous *Htt^140Q/140Q^* knock-in mouse fibroblasts and *HTT* siRNA knockdown in HeLa cells has a significant impact on protein secretion. In heterozygous HD human fibroblasts (*HTT^+/180Q^*), one copy of wild-type huntingtin is sufficient to support cargo delivery from the ER to the plasma membrane.

**Implications and future directions**

It is well-established that HD progression is linked to the toxic accumulation of mutant huntingtin protein; however, loss of wild-type huntingtin function might also contribute to neuronal cell death. Here, the authors demonstrate the requirement for wild-type huntingtin at several steps along the secretory pathway. Although in heterozygous HD human fibroblasts one copy of wild-type huntingtin is sufficient to maintain protein secretion, in neuronal cells the distances for cargo transport from the ER to the Golgi and then to the plasma membrane are extremely long. Thus, a slight impairment in the efficiency of cargo delivery is likely to result in major defects in protein secretion, which might contribute to the reduced BDNF secretion observed in HD. This study suggests a crucial function for wild-type huntingtin in the secretory pathway that cannot be fulfilled by the mutant protein and, therefore, these results could have important implications for therapies that aim to reduce huntingtin levels.

**Fig. 1.**
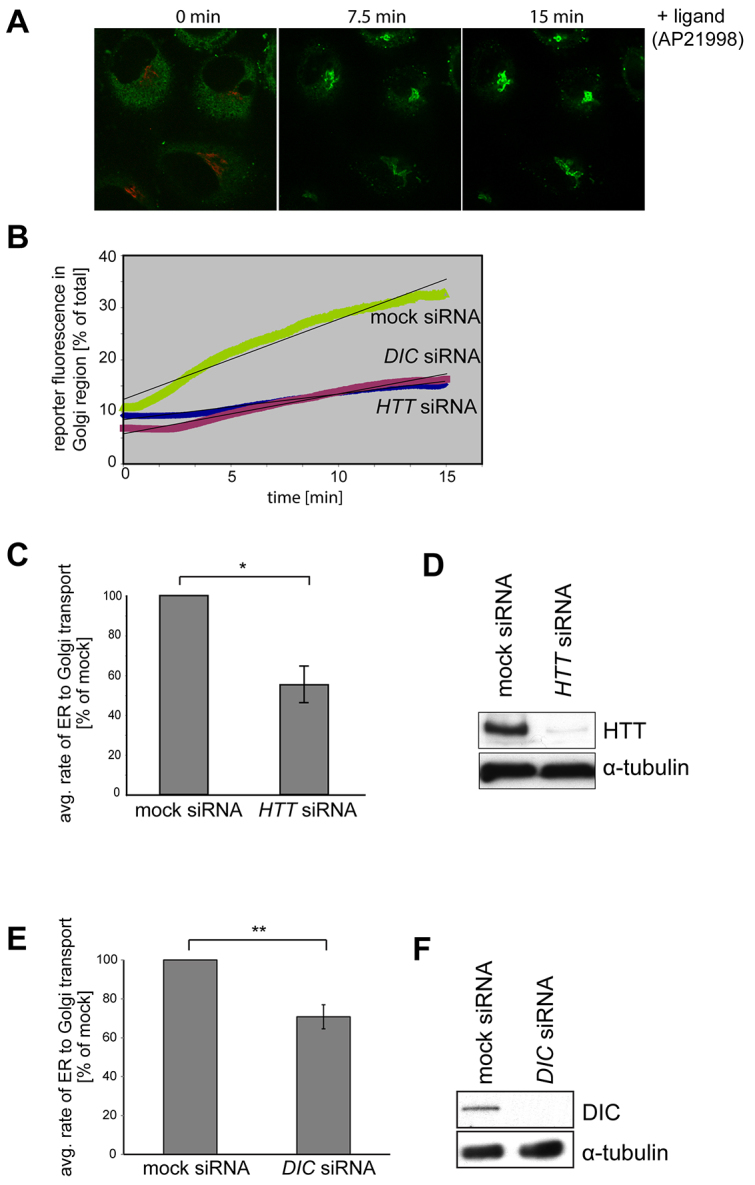
**Depletion of huntingtin and its binding partner dynein intermediate chain (DIC) reduces the rate of ER-to-Golgi transport.** (A) To follow transport of the GFP reporter from the ER to Golgi, HeLa C1 cells stably expressing the GFP-hGH reporter construct (green) and transiently transfected with GalT-mRFP (red) to mark the Golgi were fixed at the indicated times after AP21998 ligand addition and processed for immunofluorescence microscopy. (B) To analyse the rate of reporter movement from ER to Golgi, HeLa C1 cells that were either mock-treated or treated with *HTT* or *DIC* siRNA were imaged by live-cell spinning-disk microscopy after ligand addition. Volocity imaging software was used to calculate the rate of accumulation of GFP reporter in the Golgi region over time in these cells. (C) Graph depicting the average rate of ER-to-Golgi reporter transport in huntingtin-depleted cells as a percentage of mock. Huntingtin depletion resulted in a ~45% decrease in ER-to-Golgi transfer rate compared with control. 59 cells from three independent knockdown experiments were analysed using the unpaired Student’s *t*-test. Values are means ± s.e.m. (D) Representative immunoblot using antibodies against huntingtin and α-tubulin as a loading control demonstrates huntingtin protein depletion. (E) Depletion of the huntingtin binding partner DIC results in a ~30% decrease in ER-to-Golgi transfer compared with control. 93 cells from five independent knockdown experiments were analysed. (F) Representative immunoblot using antibodies against DIC and α-tubulin as a loading control demonstrates DIC protein depletion. *P≤0.05; **P≤0.01.

First, we investigated the involvement of huntingtin in the delivery of newly synthesised proteins from the ER to the Golgi complex. HeLa C1 cells were either mock-treated or transfected with siRNA targeting *HTT*. After confirming huntingtin protein depletion by immunoblotting (Fig. 1D), cells were imaged using live-cell spinning-disk microscopy. The Golgi region was visualised by expressing the RFP-tagged Golgi marker GalT (beta-1,4-galactosyltransferase-I). Still movie images depicting the transfer of the GFP-hGH from the ER (0 minutes) to the Golgi (15 minutes) after AP21998 ligand addition are shown (Fig. 1A). The rate of reporter transport from the ER to the Golgi was quantified by measuring the accumulation of fluorescent reporter intensity in the Golgi region as a percentage of the total fluorescence intensity over time (Fig. 1B). Interestingly, huntingtin depletion led to a significant reduction (~45%) in ER-to-Golgi transport (Fig. 1C), when compared with mock control cells, suggesting a kinetic delay rather than a complete block of cargo transfer from the ER to the Golgi (see supplementary material Movie 1). Deconvolving of the siRNA SMARTpool against *HTT* revealed that the individual siRNA oligo 2 leads to efficient knockdown (similar to the SMARTpool) resulting in a significant reduction in ER-to-Golgi transport (see supplementary material Fig. S1).

Dynein, a microtubule-based motor protein and huntingtin-interacting protein, has been shown to mediate cargo transport between the ER and Golgi complex (Gupta et al., 2008; Palmer et al., 2009; Presley et al., 1997; Roghi and Allan, 1999). To determine whether compromising dynein activity affects the ER-to-Golgi GFP-hGH transport assay, we performed siRNA knockdown of the dynein intermediate chain (DIC), which mediates huntingtin-dynein interaction. Loss of dynein intermediate chain causes a 35–40% reduction in transport of GFP-hGH from the ER to the Golgi complex (Fig. 1B,E). This is comparable to the results observed in cells treated with *HTT* siRNA and suggests that huntingtin and dynein might form a functional complex that mediates secretory vesicle transport along microtubules in the early secretory pathway.

### Huntingtin is involved in vesicle delivery from the Golgi complex to the plasma membrane

**Fig. 2.**
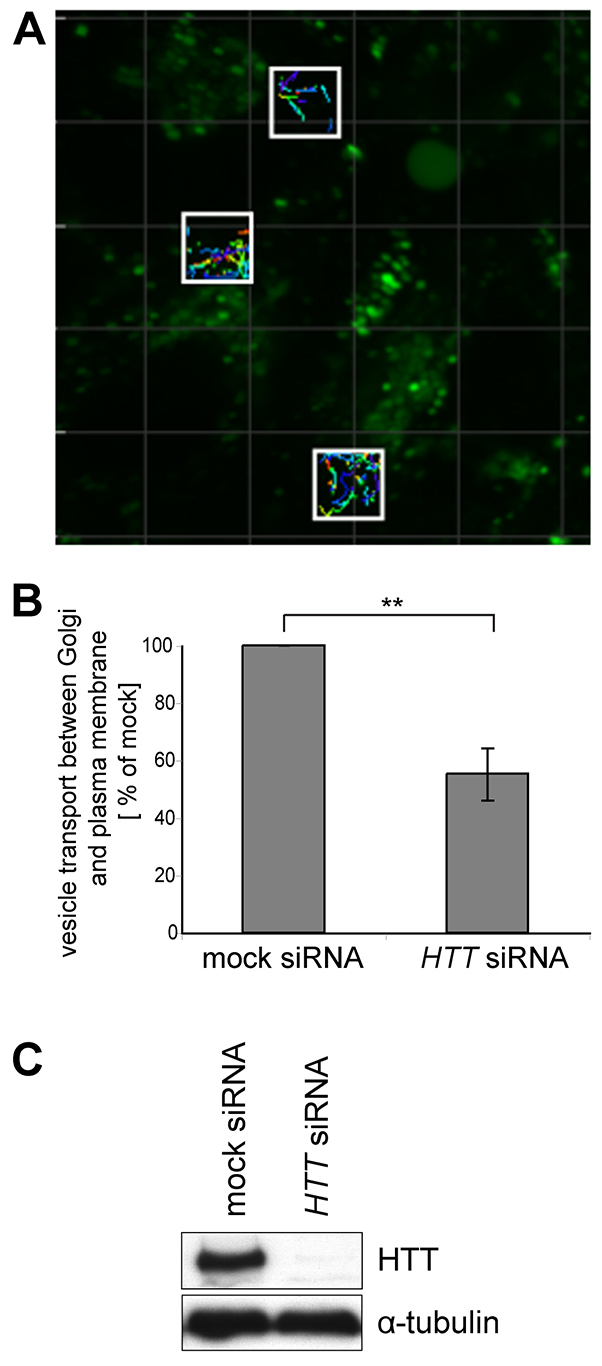
**Huntingtin depletion leads to a reduction in the number of vesicles travelling between the Golgi and plasma membrane.** (A) In HeLa C1 cells treated with *HTT* siRNA, live-cell spinning-disk microscopy was used to capture continuous 1-minute movies at defined intervals between 25 and 59 minutes after ligand addition. The total number of vesicles travelling through ten 6×6-μm areas (white boxes) between the Golgi complex and the plasma membrane was quantified over the course of 18 one-minute movies using Imaris vesicle-tracking software. (B) Analysis of 1800 regions of >630 cells from five independent knockdown experiments indicates that huntingtin depletion results in reduced numbers of secretory vesicles leaving the Golgi when compared with mock-treated cells. (C) Representative immunoblot demonstrating huntingtin protein knockdown in HeLa C1 cells. **P≤0.01.

To determine whether huntingtin depletion affects the number of GFP-hGH-reporter-containing vesicles leaving the Golgi, a series of 1-minute, confocal spinning-disk time-lapse movies was used to quantify the number of cargo-filled vesicles travelling through defined 6×6-μm regions between the trans-Golgi network and the plasma membrane (Fig. 2A). siRNA knockdown of huntingtin (Fig. 2C) led to a ~40% reduction in the number of secretory vesicles leaving the Golgi apparatus when compared with mock-treated cells (Fig. 2B). Previously, we showed that ER-to-Golgi transport is not a rate-limiting step in the overall secretory process and a kinetic delay in this early secretory pathway does not always have a knock-on effect on post-Golgi trafficking (Bond et al., 2011). Thus, we propose a direct role for huntingtin in the formation of secretory vesicles at the trans-Golgi network.

### Depletion of huntingtin reduces the number of exocytic vesicle fusion events at the plasma membrane

**Fig. 3.**
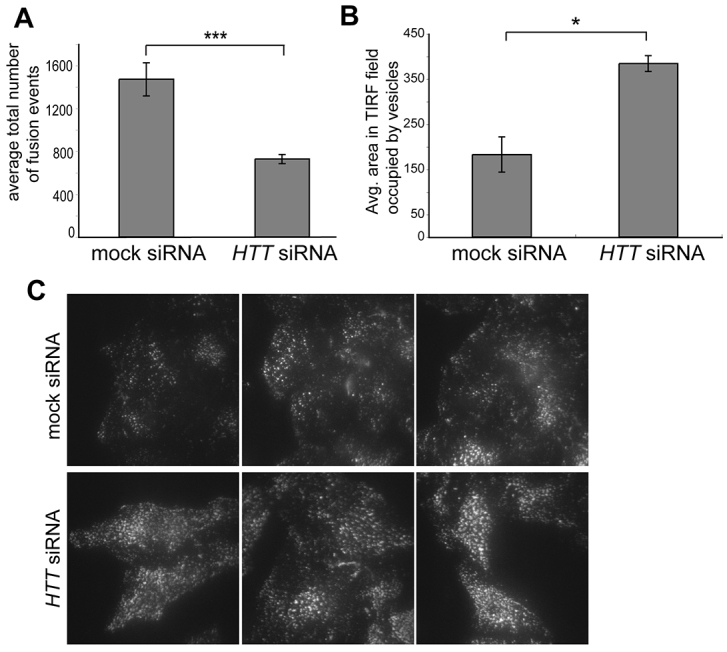
**Huntingtin loss reduces the number of vesicle fusion events and increases the number of docked vesicles at the plasma membrane.** (A) Vesicle fusion events in mock or huntingtin-depleted HeLa C1 cells were monitored by live-cell TIRF microscopy at 5-minute intervals between 25 and 59 minutes after ligand addition. Huntingtin depletion causes a significant decrease of 50% in average number of vesicle fusion events at the plasma membrane. 170 cells from four independent knockdown experiments performed in duplicate or triplicate were analysed using the Student’s *t*-test; values are means ± s.e.m. (B) In mock or huntingtin-depleted HeLa C1 cells, the total area in the TIRF field occupied by fluorescent vesicles in single-plane images taken at specific intervals between 25 and 60 minutes after AP21998 ligand addition was calculated using Volocity analysis software. Huntingtin knockdown results in a significant increase in the area occupied by docked vesicles as compared with mock. 170 cells from four independent knockdown experiments (as used in the TIRF fusion assay) were analysed using the Student’s *t*-test; values are means ± s.e.m. (C) Vesicles in the TIRF field at the base of mock and huntingtin-depleted cells. *P≤0.05; ***P≤0.001.

To examine the role of huntingtin at the plasma membrane in live cells, we used TIRF (total internal reflection fluorescence) microscopy, which allows selective imaging of a ~100 nm region beneath the plasma membrane. Due to the high signal-to-noise ratio, single fusion events of fluorescent secretory cargo vesicles with the plasma membrane can be visualised as a short flash in the TIRF field. Loss of wild-type huntingtin resulted in a significant decrease of ~50% in the total number of fusion events as compared with control cells (Fig. 3A, see supplementary material Movie 2) and an observed accumulation of secretory vesicles underneath the plasma membrane (Fig. 3B). Indeed, quantitation of the total area in the TIRF field occupied by secretory vesicles captured at intervals between 25 and 59 minutes after ligand addition revealed a significant increase in docked vesicles in huntingtin-knockdown cells when compared with mock-treated cells (Fig. 3B,C). These results clearly demonstrate a direct role for huntingtin in the final fusion events during exocytosis.

### Homozygous primary *Htt^140Q/140Q^* MEFs, but not heterozygous HD patient fibroblasts, have a delay in cargo transport between the ER and Golgi

**Fig. 4.**
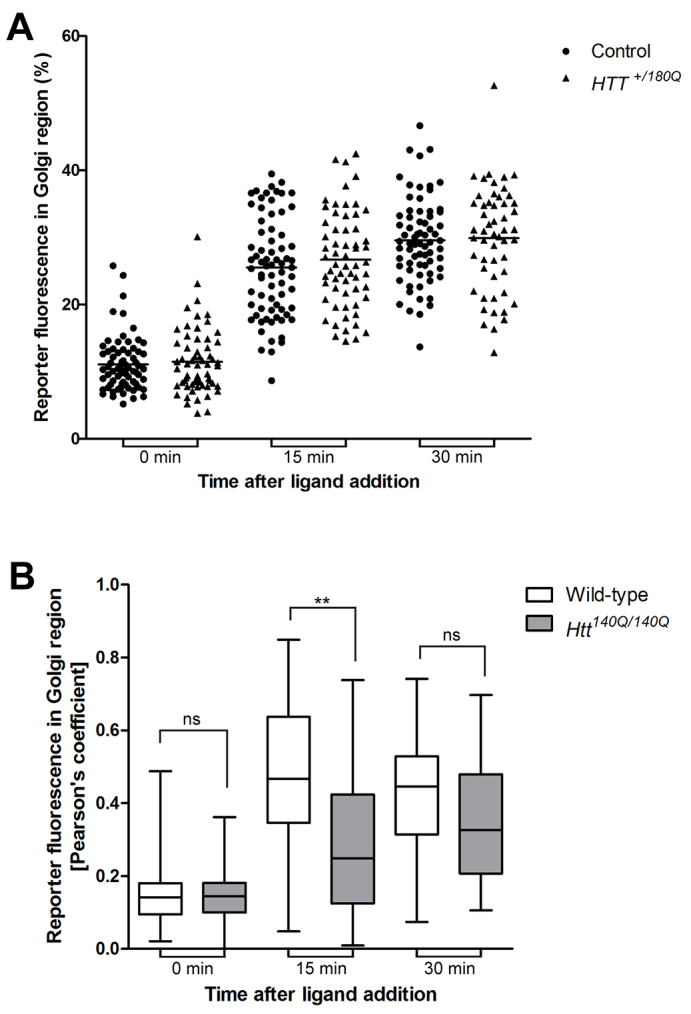
**ER-to-Golgi transport of a fluorescent reporter is reduced in homozygous *Htt^140Q/140Q^* KI MEFs but not in heterozygous patient fibroblasts with a 180Q expansion.** (A) Human fibroblasts from a healthy control and a patient with juvenile-onset HD (*HTT^+/180Q^*) were transiently transfected with the GFP-hGH reporter construct. Cells were untreated or treated with the ligand AP21998 for 0, 15 and 30 minutes, fixed and stained with antibodies to GFP and the Golgi protein GM130. The amount of fluorescent reporter present in the Golgi region was quantified in confocal images using Volocity image-analysis software in >350 cells from four independent experiments and is illustrated as a scatter plot with the line representing the mean. There was no significant difference in reporter movement from the ER to Golgi in HD patient fibroblasts compared with control. (B) Mouse embryonic fibroblasts (MEFs) were isolated from wild-type and *Htt^140Q/140Q^* knock-in mice at E17/18 and transiently transfected with the GFP-hGH reporter construct. At 0, 15 and 30 minutes after AP21998 ligand addition, GFP and GM130 colocalisation images were analysed by calculating the Pearson’s coefficient using Volocity image-analysis software. Compared with wild-type MEFs, significantly less cargo accumulated in the Golgi of homozygous *Htt^140Q/140Q^* knock-in MEFs in >120 cells in four independent experiments and is illustrated as a box-and-whisker plot. The box represents the median, 25th and 75th percentiles and whiskers represent the maximum and minimum. A one-way ANOVA followed by a post-hoc Bonferroni multiple comparison test was conducted as a statistical test. **P≤0.01. ns, not significant.

Finally, we analysed the secretory pathway in human fibroblasts from a healthy control (GM23976A) and an individual with juvenile-onset HD [heterozygous for an expanded polyQ tract of 180Q (*HTT^+/180Q^*); GM09197B], and in primary MEFs isolated from wild-type mice (typically around E17/18) and homozygous *Htt^140Q/140Q^* KI mice. The human and mouse fibroblasts were transiently transfected with the GFP-hGH reporter construct and the amount of this reporter molecule in the Golgi area was analysed at 0, 15 and 30 minutes after AP21998 ligand addition. As shown in Fig. 4A, there was no significant difference in reporter movement from the ER to the Golgi in control compared with heterozygous *HTT^+/180Q^* patient fibroblasts. However, significantly less cargo accumulated in the Golgi 15 minutes after ligand addition in homozygote *Htt^140Q/140Q^* KI MEFs when compared with wild-type MEFs (Fig. 4B). Thus, efficient cargo transport in the early secretory pathway requires at least one copy of wild-type huntingtin, which cannot be replaced by the overexpression of mutant 140Q huntingtin in primary MEFs. At least in fibroblasts, the presence of one copy of mutant huntingtin with a 180Q tract does not seem to disrupt cargo transport in the secretory pathway.

## DISCUSSION

Huntingtin has multiple roles in cellular membrane trafficking and associates with microtubule-based kinesin and dynein motor proteins, but is also linked via its interacting protein optineurin with the actin-associated motor myosin VI (Hattula and Peränen, 2000; Sahlender et al., 2005; Bond et al., 2011). Using a microscopy-based secretion assay in HeLa cells, we have established that huntingtin is required for precise stages of protein secretion: for ER-to-Golgi transport as well as vesicle fusion at the cell surface. In addition, our data suggest that huntingtin might be required for the formation of exocytic vesicles at the trans-Golgi complex and trafficking of vesicles from the Golgi to the plasma membrane. Dynein has also been implicated in the trafficking between the ER and Golgi complex (Lee et al., 2004; Lippincott-Schwartz et al., 1995; Stauber et al., 2006) and kinesin motors for post-Golgi transport to the plasma membrane (Kreitzer et al., 2000). Huntingtin interacts with dynein and kinesin via HAP1 (Harjes and Wanker, 2003; Li and Li, 2004) and directly binds to dynein intermediate chain (Caviston et al., 2007); thus, huntingtin might function as a key factor along the secretory pathway by promoting the association of vesicles with the cytoskeleton and by acting as a molecular switch via its respective interactions with these microtubule-based motor proteins.

Huntingtin function in the final fusion events at the plasma membrane could involve its binding partner optineurin, a myosin-VI-associated cargo adaptor. The importance of a functional complex of huntingtin, myosin VI and optineurin during vesicle fusion at the plasma membrane is supported by the fact that the absence of any of these proteins results in a very similar phenotype: a reduction in the number of vesicle fusion events at the plasma membrane and an accumulation of vesicles beneath the plasma membrane (Bond et al., 2011; and this work).

HeLa C1 cells are an ideal system to visualise cargo transport in the secretory pathway. The movement of large, bright exocytic vesicles between the ER and Golgi complex (see supplementary material Movie 2) and then to the plasma membrane can be followed by live-cell microscopy. The large size and content of fluorescent cargo in stable HeLa C1 cells also enables one to visualise and quantify single secretory cargo fusion events at the plasma membrane (see supplementary material Movie 2). However, in primary MEFs isolated from the *Htt^140Q/140Q^* KI mouse or in human patient fibroblasts, single cargo vesicles are smaller and contain much less fluorescent content, making it challenging to analyse the movement of single exocytic vesicles and to image distinct post-Golgi cargo vesicles as well as single fusion events at the plasma membrane in the TIRF field.

The crucial role of huntingtin in cargo transport in the early secretory pathway (see Figs 1 and 4) was clearly demonstrated by loss of wild-type huntingtin in HeLa cells after siRNA knockdown and also by expression of only mutant 140Q huntingtin in primary homozygous MEFs, leading to a kinetic delay in the movement of cargo from the ER to the Golgi complex. Interestingly, fibroblasts from a heterozygous individual with HD (*HTT^+/180Q^*), which still express one wild-type copy of huntingtin, showed no significant defect in the secretory pathway. The presence of one copy of wild-type huntingtin seems to be sufficient for maintaining secretion, at least in fibroblasts. In addition, the wild-type protein could also have a protective role with respect to the defects caused by expression of the mutant protein, as shown in a *Drosophila* model of HD (Arribat et al., 2013). Moreover, the ratio of wild-type to mutant huntingtin might be crucial in determining cell fate (Saleh et al., 2014) and, thus, strategies to reduce mutant huntingtin levels are being developed (Stanek et al., 2014).

In neuronal cells, in contrast to fibroblasts, the distances for cargo to be transported between the ER, Golgi and plasma membrane are extremely large and, therefore, a slight impairment in the efficiency of delivery is likely to result in major defects in protein secretion, which might contribute to the disease pathology associated with reduced BDNF secretion in HD (Wang et al., 2012; Zala et al., 2008; Pineda et al., 2009; Gauthier et al., 2004). Neuronal membrane trafficking is believed to occur via the same basic membrane compartments as those present in non-neuronal cells; however, the polarised nature of nerve cells and the distinct morphology of long cell projections in the form of axons and dendrites is linked to a more complex organisation and distribution of these compartments. We tested whether the reporter molecule GFP-hGH could be used to measure the rate of exocytosis in primary cortical neurons but, due to the toxicity of the reporter construct and the specialised neuronal morphology, it was extremely difficult to measure the rate of transport between the ER and the Golgi complex.

In summary, this novel regulated exocytosis assay has allowed us to demonstrate the requirement for huntingtin at several steps along the secretory pathway. In fibroblasts, the complete absence of wild-type huntingtin in homozygous *Htt^140Q/140Q^* KI MEFs and after *HTT* siRNA knockdown in HeLa cells reduces protein secretion. In fibroblasts from a heterozygous individual with HD (*HTT^+/180Q^*), one copy of huntingtin is sufficient to support cargo delivery in the exocytic pathway. The majority of cell types are most likely able to compensate for a slight reduction in secretory efficiency; however, the striatal neurons most affected in HD might be particularly susceptible to alterations in the trafficking of cargo, such as receptors and transporters that are vital for neuronal cell survival. This study suggests a crucial function for wild-type huntingtin that cannot be fulfilled by the mutant protein and, thus, these results could have important implications for therapies that aim to reduce levels of mutant huntingtin.

## MATERIALS AND METHODS

### Antibodies

The following antibodies were used: anti-huntingtin (1HU-4C8; Millipore); anti-optineurin (Sahlender et al., 2005); anti-GFP (Invitrogen); anti-GM130 (BD Biosciences); anti-DIC (MAB1618; Chemicon); a mouse monoclonal to tubulin (Sigma).

### Constitutive secretion assay

The constitutive secretion assay was generated by modifying the RPD Regulated Secretion/Aggregation Kit (Ariad Pharmaceuticals, Cambridge, MA) as described previously (Gordon et al., 2010; Bond et al., 2011). A stable HeLaM cell line, termed HeLa C1, was generated and single clones with moderate expression levels of the secretory cargo were selected for further use. Secretion of the reporter construct was initiated by addition of 1 μM AP21998, a ligand that binds to the aggregation domains and thereby solubilises the reporter molecule and promotes exit from the ER.

### Cell culture, transfection and siRNA

The stable HeLa C1 cell line was grown in Dulbecco’s modified Eagle medium (DMEM; Sigma, UK) supplemented with 10% fetal bovine serum (FBS), 2 mM L-glutamine, penicillin/streptomycin (Sigma-Aldrich, UK) and puromycin (1.66 μg/ml) at 37°C and 5% CO_2_. Human fibroblasts from a healthy control (GM23976) and an individual with juvenile-onset HD with a 180Q expansion (Sathasivam et al., 1997) (GM09197; *HTT^+/180Q^*) were obtained from Coriell Cell Repositories and cultured in Minimum Essential Medium (MEM) with Earl’s salts, non-essential amino acids, and sodium bicarbonate (M5650; Sigma) supplemented with 15% FBS and 2 mM L-glutamine at 37°C and 5% CO_2_. Mouse embryonic fibroblasts (MEFs) were established from wild-type and *Htt^140Q/140Q^* embryos at day E17/18 of development. For preparation of fibroblasts, skin, limbs and muscle tissue was dissected from the embryos, minced and incubated for 20 min at 37°C with trypsin containing 300 U/ml DNase (Invitrogen). After addition of growth medium (DMEM with 10% FBS, glutamine, penicillin and streptomycin), the digested tissue was dissociated using a glass Pasteur pipette, centrifuged, resuspended in growth media and plated onto tissue-culture treated dishes.

All siRNA oligos were obtained from Dharmacon. ON-TARGETplus SMARTpool oligos (or individual siRNA oligos 1 and 2 against *HTT*) were used to knock down huntingtin or dynein intermediate chain. For efficient knockdown, HeLa C1 cells were transfected twice with four siRNA oligos on day 1 and 3 using OligofectAMINE (Invitrogen). On day 5, cells were processed for corresponding assays and the efficiency of protein depletion was assessed by western blotting.

HeLa C1 cells were transfected with the GalT-mRFP plasmid (provided by Dr G. Patterson, Lippincott-Schwartz Lab, National Institute of Child Health and Human Development, MD) using FuGENE 6 (Roche Diagnostics) according to the manufacturer’s instructions.

### Immunofluorescence

For immunofluorescence, primary MEFs, human fibroblasts or HeLa C1 cells were grown on coverslips, transfected with the corresponding plasmids and, 24 hours later, were fixed with 4% PFA, permeabilised with 0.1% Triton X-100, blocked with 1% BSA in PBS and processed for indirect immunofluorescence using primary antibodies (specified in the figure legends) and secondary antibodies coupled with Alexa Fluor 488 or Alexa Fluor 568 (Molecular Probes). After mounting, cells were imaged using a Zeiss LSM710 confocal microscope.

### Spinning-disk microscopy and TIRF microscopy

For live-cell TIRF or spinning-disk microscopy studies, cells were grown on 25-mm round coverslips (VWR International, UK) and imaged at 37°C in CO_2_-independent medium (Invitrogen, UK). Images were obtained on a Zeiss microscope equipped with spinning disk and TIRF attachment (Carl Zeiss, Inc., UK). Images were acquired with a Hamamatsu Photonics EM-CCD Digital Camera (Hamamatsu, Japan) and Zeiss Zen imaging software (Carl Zeiss, Inc., UK). Volocity analysis software (Improvision, UK) was used to quantify the rate of transfer of the reporter molecule from the ER to the Golgi complex in spinning-disk videos. Imaris vesicle tracking software (Bitplane) was used to quantify the total number of vesicles travelling through 6×6-μm areas between the Golgi complex and the plasma membrane.

## Supplementary Material

Supplementary Material
