# Supplementary material for: Huntingtin is required for ER-to-Golgi transport and for secretory vesicle fusion at the plasma membrane
Source: Dis Model Mech. 2014 Oct 31;7(12):1335–40. doi: 10.1242/dmm.017368 (PMC4257002; doi:10.1242/dmm.017368)
Supplement: Supplementary Material [file supp_7_12_1335__index.html]

Huntingtin is required for ER-to-Golgi transport and for secretory vesicle fusion at the plasma membrane — Supplementary Material 

# Huntingtin is required for ER-to-Golgi transport and for secretory vesicle fusion at the plasma membrane

## DMM017368 Supplementary Material

**Files in this Data Supplement:**

- **Supplementary Material**
