# Supplementary material for: Huntingtin is required for ER-to-Golgi transport and for secretory vesicle fusion at the plasma membrane
Source: Dis Model Mech. 2014 Oct 31;7(12):1335–40. doi: 10.1242/dmm.017368 (PMC4257002; doi:10.1242/dmm.017368)
Supplement: Supplementary Material [file supp_7.12.1335_DMM017368.pdf]

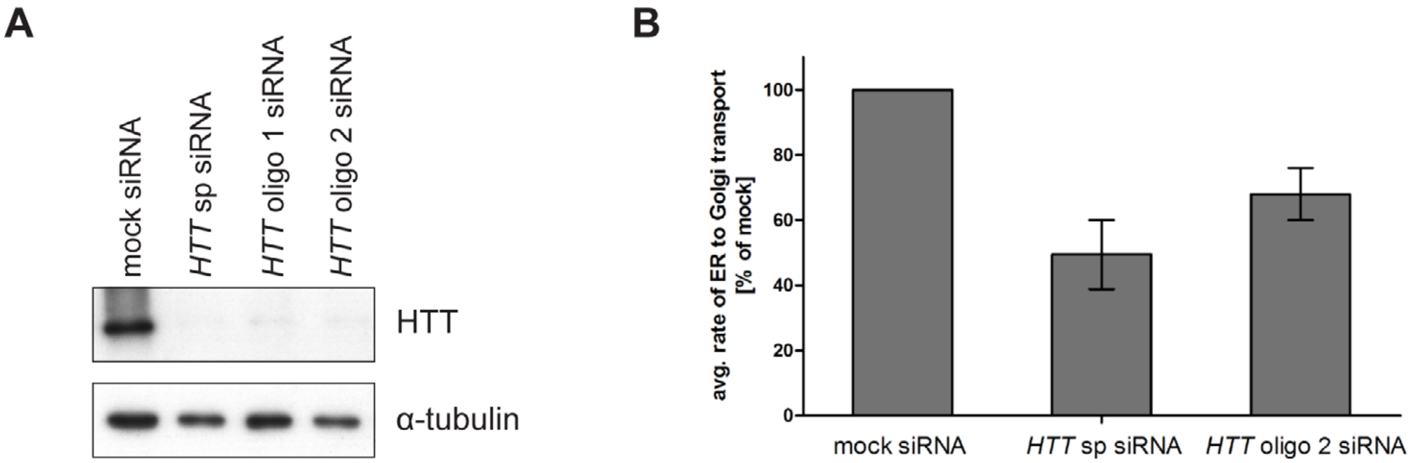

Fig. S1. Individual siRNA oligos against huntingtin reduce the rate of reporter movement from ER to Golgi. (A) Two individual siRNA oligos (oligo 1 and 2) against huntingtin were compared to the SMARTpool (sp) for their ability to deplete huntingtin protein levels. Representative immunoblot using antibodies against huntingtin and α-tubulin as loading control demonstrates efficient huntingtin protein depletion by the SMARTpool siRNA and also by the individual siRNA oligos. **(B)** To analyse the rate of reporter movement from ER to Golgi, HeLa C1 cells either mock, *HTT* SMARTpool or *HTT* oligo 2 siRNA treated were imaged by live-cell spinning-disk microscopy after ligand addition. Volocity image analysis software was used to calculate the rate of accumulation of GFP reporter in the Golgi region over time in these cells. Graph depicting the average rate of ER to Golgi reporter transport in huntingtin-depleted cells as a percentage of mock. Huntingtin depletion by siRNA SMARTpool results in a similar reduction in ER-to-Golgi transfer rate as observed after depletion using oligo 2 siRNA. 21 cells from one representative knockdown experiment were analyzed. Values are means ± s.e.m.

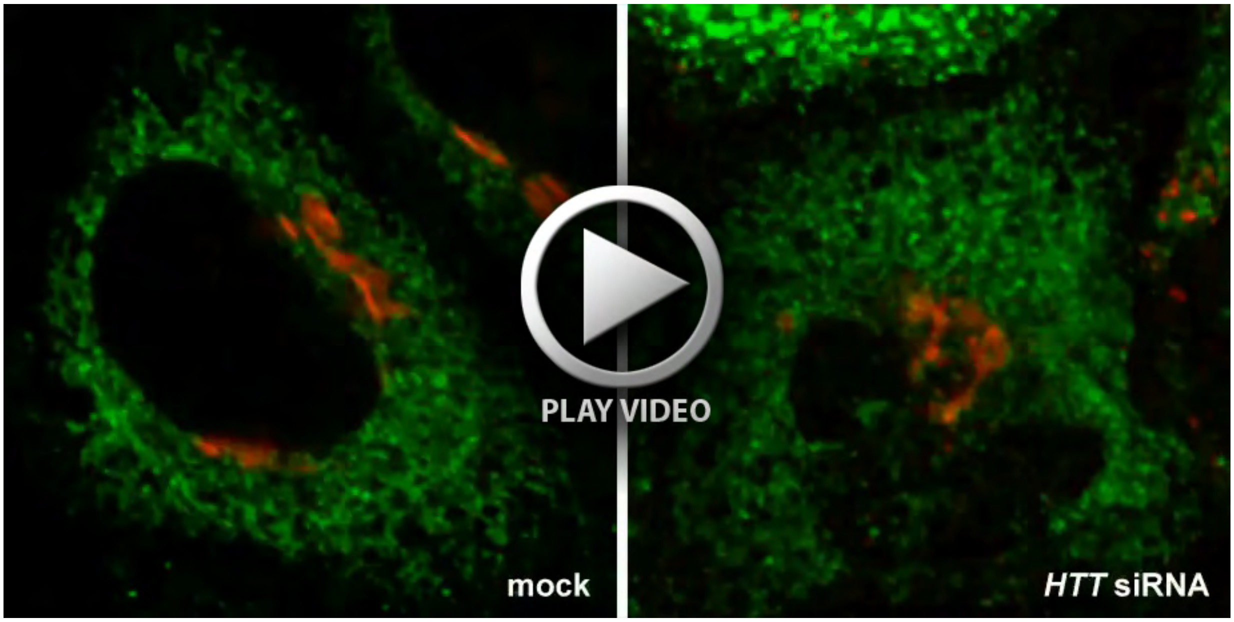

Movie 1. Reporter movement from ER to Golgi of mock treated and huntingtin depleted cells. HeLa C1 cells either mock or *HTT* siRNA treated were imaged by live-cell spinning-disk microscopy after ligand addition.

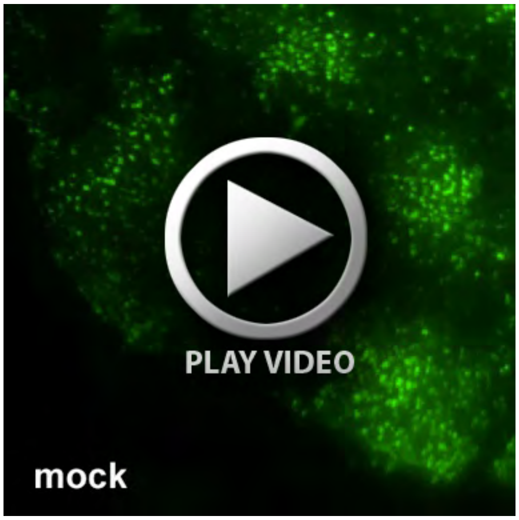

Movie 2. Vesicle fusion events at the plasma membrane of mock treated cells. Vesicle fusion events seen as flashes in mock treated HeLa C1 cells monitored by live cell TIRF microscopy at least 25 min after ligand addition.
